# Effect of Surgeons’ Years of Experience on Outcomes of Acute Type A Aortic Dissection

**DOI:** 10.7759/cureus.75499

**Published:** 2024-12-10

**Authors:** Baku Takahashi, Keiji Kamohara, Hiroyuki Morokuma, Sojiro Amamoto

**Affiliations:** 1 Department of Thoracic and Cardiovascular Surgery, Faculty of Medicine, Saga University, Saga, JPN

**Keywords:** aortic dissection, aortic team, assistant’s experience, surgeons’ experience, surgeons’ years in practice

## Abstract

Background

The effect of surgeons’ years of experience on the outcomes of acute type A aortic dissection (ATAAD) repair has not yet been studied. This study aimed to evaluate the association between the surgeon’s years in practice and the outcomes of ATAAD repair.

Methods

Surgical records of ATAAD repairs performed at Saga University Hospital between 2004 and 2020 were reviewed. Surgeons were divided into two groups based on their surgical experience: late-career surgeons (LCSs) and early-career surgeons (ECSs) with ≥16 years and <16 of practice, respectively. The surgeons were designated as the primary surgeons or first assistants and grouped as follows: LCS-LCS, LCS-ECS, ECS-LCS, and ECS-ECS.

Results

During the study period, 25 primary surgeons performed 203 ATAAD repairs with 31 different first assistants: LCS-LCS, 50 repairs; LCS-ECS, 82 repairs; ECS-LCS, 55 repairs; and ECS-ECS, 16 repairs. The mean years in practice as a primary surgeon was 19.8 ± 3.3 for LCSs and 13.0 ± 1.8 for ECSs (p < 0.01). The unadjusted in-hospital mortality rates were 10.0%, 12.2%, 5.5%, and 6.3% for the LCS-LCS, LCS-ECS, ECS-LCS, and ECS-ECS groups, respectively (p = 0.63). Multivariable regression analysis showed that the surgeon’s years of experience in practice were not a risk factor for in-hospital mortality. Furthermore, the long-term survival rate did not differ between the groups (p = 0.62).

Conclusions

The surgeons’ years in practice had no effect on the outcomes of ATAAD repair. These investigations could aid in on-call coverage for ATAAD in medium-sized centers.

## Introduction

To improve surgical results, it is important to evaluate the relationship between surgeons’ experience and surgical outcomes. Some studies have reported that surgeons’ age [1-6] and surgical volume [7-9] affected surgical results. Similarly, surgeons’ years in practice have recently gained attention [10,11] because the length of practice is considered to precisely measure surgeons’ experience [2,4]. However, only a few studies on the association between surgical outcomes and surgeons’ years of experience in practice have been conducted in the field of cardiac surgery.

In limited studies, it is reported that the effect of years of experience varies with the type of surgery [10], indicating that the more complicated the surgeries, the more strongly they are affected by the surgeons’ experience. A study regarding surgery for a congenital heart condition reported that experienced surgeons in a surgical team helped inexperienced operators improve their performance [11]. Acute type A aortic dissection (ATAAD) requires immediate cardiac surgery [12,13] and has one of the highest mortality rates [8,14]. Therefore, we hypothesized that surgeons with more experience, performing as primary surgeons or even as assistants, would achieve better outcomes in ATAAD repair.

The aim of the present study was to clarify the association between the surgeons’ years in practice and the outcomes of ATAAD repair by reviewing data related to ATAAD repairs at our hospital.

## Materials and methods

Study design

All data were extracted from the medical records of Saga University Hospital (Saga Prefecture, Japan) between April 2004 and March 2020. This study was approved by the institutional review board of Saga University Hospital, and opt-out consent was obtained (2021-02-R-02). The requirement for prospective patient consent was waived because of the retrospective nature of the study. In total, 203 consecutive patients who underwent ATAAD repair were included.

Surgeons

To evaluate the impact of surgeons’ years in practice, surgeons were categorized into two groups: late-career surgeons (LCSs) and early-career surgeons (ECSs) with ≥16 years and <16 years of clinical experience, respectively. Years of clinical experience were counted from the year of graduation from the postgraduate year of medical school and the year of certification as a medical doctor. The 16-year threshold was determined based on organizational status. In Japan, there is an organization of young cardiovascular surgeons called “under 40” officially recognized by the Japanese Society for Cardiovascular Surgery (https://jscvs.or.jp/u-40/). Since age under 40 years is generally equivalent to less than 16 years of experience for doctors in Japan, we chose 16 years of experience as the cutoff. The surgeons were designated as the primary surgeons or first assistants. Therefore, there were four surgical operating teams: LCSs as the primary surgeon and the first assistant (LCS-LCS), an LCS as the primary surgeon with an ECS as the first assistant (LCS-ECS), an ECS as the primary surgeon and an LCS as the first assistant (ECS-LCS), and ECSs in both roles (ECS-ECS).

Surgery

Our standard surgical treatment strategy for ATAAD, which has been described previously [15], is based on tear-oriented surgery. However, we considered total arch replacement based on the patient's age, medical history, and clinical status. The location and number of cannulations, choice of brain protection method, degree of hypothermia, range of aortic replacement, use of the frozen elephant trunk technique, and concomitant procedures were decided on a patient-wise basis. In terms of distal anastomosis, an open distal anastomosis method was chosen for all repairs.

Statistical analyses

All statistical analyses were performed using the JMP software (version 16.0; SAS Institute, Cary, NC, USA). Continuous data were expressed as mean ± standard deviation using a t-test or one-way analysis of variance (ANOVA). Nominal and categorical variables were compared using the χ2 tests or Fisher’s exact test. Binomial logistic regression analysis for in-hospital mortality was performed on all pre- and intraoperative variables using a forward-backward stepwise approach based on a p-value of <0.1. Survival and aortic event-free rates were estimated using the Kaplan-Meier curves, and log-rank tests were used for comparisons. The p-values were considered statistically significant at 0.05.

## Results

Characteristics of surgeons and patients

The profiles of the surgeons who participated in the ATAAD repair are summarized in Table 1.

**Table 1:** Surgeons’ profile Values are expressed as n for numbers, and mean ± standard deviation (range) using a t-test for continuous variables, in which the t-values are provided. The p-values were considered statistically significant at 0.05. LCS: late-career surgeon; ECS: early-career surgeon; ATAAD: acute type A aortic dissection

	As the primary surgeon				As the first assistant			
	LCS	ECS	t-value	p-value	LCS	ECS	t-value	p-value
Number	13	12	-	-	12	19	-	-
Age (years)	44.2 ± 3.2 (40-53)	37.5 ± 1.9 (33-42)	-16.35	<0.01	47.8 ± 6.0 (40-64)	34.0 ± 4.1 (28-42)	-18.97	<0.01
Years in practice	19.8 ± 3.3 (16-29)	13.0 ± 1.8 (9-15)	-15.92	<0.01	23.4 ± 5.7 (16-37)	9.9 ± 3.0 (4-15)	-20.99	<0.01
ATAAD repair cases/year	4.7 ± 2.5 (1-9)	5.3 ± 3.1 (1-10)	1.53	0.13	2.9 ± 2.5 (0-9)	1.7 ± 2.5 (0-10)	-3.54	<0.01

During the study period, 25 primary surgeons and 31 primary assistants were involved. The mean numbers of surgeons’ years in practice were 19.8 ± 3.3 years (range, 16-29) for LCSs and 13.0 ± 1.8 years (range, 9-15) for ECSs as primary surgeons (p < 0.01), and 23.4 ± 5.7 years (range 16-37) for LCSs and 9.9 ± 3.0 years (range, 4-15) for ECSs as first assistants (p < 0.01). The mean annual surgical volume of ATAAD repairs of LCSs and ECSs as primary surgeons was 4.7 ± 2.5 and 5.3 ± 3.1 cases/year, respectively (p = 0.13). During the study period, 203 ATAAD repairs were performed: LCS-LCS, 50 repairs; LCS-ECS, 82 repairs; ECS-LCS, 55 repairs; and ECS-ECS, 16 repairs. In our hospital, the annual average ATAAD surgical volume was 12.7 ± 5.4 cases/year. Although the LCSs operated on all patients with a history of open-heart surgery and the surgeries performed by the ECS-ECS group tended to have a lower risk of comorbidities, there was no significant difference in preoperative variables between all surgical teams (Table 2).

**Table 2:** Preoperative data Values are presented as n (%) for categorical variables or mean ± standard deviation for continuous variables. Continuous variables were compared using one-way analysis of variance (ANOVA), and the F-values were provided. Categorical variables were compared using either the χ² test or Fisher’s exact test; where applicable, chi-square values are reported. For variables analyzed using Fisher’s exact test, no test statistic is provided. A p-value < 0.05 was considered statistically significant. LCS-LCS: late-career surgeons as the primary surgeon and the first assistant; LCS-ECS: a late-career surgeon as the primary surgeon and an early surgeon as the first assistant; ECS-LCS: an early-career surgeon as the primary surgeon and a late-career surgeon as the first assistant; ECS-ECS: early-career surgeons as both the primary surgeon and the first assistant; AF: atrial fibrillation; CAD: coronary artery disease; CVD: cerebrovascular disease; HD: hemodialysis; AR: aortic valve regurgitation

Admission variables	LCS-LCS	LCS-ECS	ECS-LCS	ECS-ECS	Chi-square value	F- value	P-value
Number of patients (n, %)	50 (24.6)	82 (40.4)	55 (27.1)	16 (7.9)	-	-	-
Patient age (mean ± SD)	64.7 ± 13.9	67.5 ± 11.8	70.7 ± 14.1	68.2 ± 9.6	-	1.91	0.13
Male	26 (56.0)	36 (43.9)	26 (47.3)	11 (68.8)	3.59	-	0.31
Hypertension	38 (76.0)	62 (75.6)	38 (69.1)	12 (75.0)	0.91	-	0.82
Diabetes	5 (10.0)	2 (2.4)	4 (7.3)	1 (6.3)	-	-	0.22
Chronic AF	0	1 (1.2)	3 (5.5)	1 (6.3)	-	-	0.18
History of CAD	5 (10.0)	4 (4.9)	1 (1.8)	3 (18.8)	-	-	0.06
History of CVD	7 (14.0)	12 (14.6)	7 (12.7)	0	-	-	0.49
History of open heart surgery	5 (10.0)	4 (4.9)	0	0	-	-	0.08
Creatinine (mg/dL)	1.1 ± 1.1	1.3 ± 1.4	1.1 ± 1.2	1.5 ± 1.8	-	0.50	0.68
HD	1 (2.0)	2 (2.5)	1 (1.8)	1 (6.3)	-	-	0.68
Marfan syndrome	1 (2.0)	0	0	0	-	-	0.33
Shock vital	13 (26.0)	19 (23.2)	17 (30.9)	3 (18.8)	1.47	-	0.69
Tamponade	8 (16.0)	14 (17.1)	11 (20.0)	1 (6.3)	1.71	-	0.64
Rupture	2 (4.0)	0	1 (1.8)	0	-	-	0.25
AR≥moderate	3 (6.0)	6 (7.3)	1 (1.8)	3 (18.8)	-	-	0.11
Acute neurological complication	5 (10.0)	7 (8.5)	6 (10.9)	4 (25.0)	3.81	-	0.30
Malperfusion	17 (34.0)	22 (26.8)	15 (27.3)	6 (37.5)	1.69	-	0.63
Acute mesenteric ischemia	3 (6.0)	3 (3.8)	0	0	-	-	0.34

Intraoperative variables

All intraoperative variables are listed in Table 3. The LCS-ECS group was significantly less likely to perform ascending aortic replacement or hemiarch replacement than the other groups (p = 0.01). Although there was no statistical difference, the LCS-LCS group performed more aortic root procedures (p = 0.40), and the ECSs as the primary surgeons used the frozen elephant trunk technique more frequently. There was no significant difference in concomitant procedures between the surgical groups (p = 0.96). Although the ECSs as the primary surgeons had significantly shorter surgical operative (p < 0.01) and cardiopulmonary bypass times (p = 0.05), there was no difference in circulatory arrest times. The LCS-LCS group used significantly less selective antegrade cerebral perfusion (p = 0.01) and had a lower rectal minimum temperature (p < 0.01). Red blood cell and platelet transfusions were more likely to be performed when LCSs were the primary surgeons.

**Table 3:** Intraoperative data Values are presented as n (%) for categorical variables or mean ± standard deviation for continuous variables. Continuous variables were compared using one-way analysis of variance (ANOVA), and the F-values were provided. Categorical variables were compared using either the χ² test or Fisher’s exact test; where applicable, chi-square values are reported. For variables analyzed using Fisher’s exact test, no test statistic is provided. A p-value < 0.05 was considered statistically significant. LCS-LCS: late-career surgeons as the primary surgeon and the first assistant; LCS-ECS: a late-career surgeon as the primary surgeon and an early-career surgeon as the first assistant; ECS-LCS: an early-career surgeon as the primary surgeon and a late-career surgeon as the first assistant; ECS-ECS: early-career surgeons as both the primary surgeon and first assistant; AAR: ascending aortic replacement; TAR: total arch replacement; CABG: coronary artery bypass grafting; AVR: aortic valve replacement; CPB: cardiopulmonary bypass; CA: circulation arrest; ACP: antegrade cerebral perfusion; RBC: packed red blood cell; FFP: fresh frozen plasma

Admission variables	LCS-LCS (n = 50)	LCS-ECS (n = 82)	ECS-LCS (n = 55)		ECS-ECS (n = 16)	Chi-square value	F-value	p-value
AAR or hemiarch replacement (n, %)	35 (70.0)	43 (52.4)		43 (78.2)	12 (75.0)	11.19	-	0.01
TAR	14 (28.0)	37 (45.1)		11 (20.0)	4 (25.0)	10.7	-	0.01
Partial arch replacement	1 (2.0)	2 (2.4)		1 (1.8)	0	-	-	1.00
Entry closure	40 (80.0)	67 (81.7)		52 (94.6)	14 (87.5)	5.75	-	0.12
Frozen elephant trunk	6 (12.0)	8 (9.8)		11 (20.0)	4 (25.0)	4.55	-	0.21
Aortic root procedure	9 (18.0)	9 (11.0)		4 (7.3)	2 (12.5)	2.99	-	0.40
Concomitant procedures	8 (16.0)	12 (14.6)		8 (14.6)	3 (18.8)	0.22	-	0.96
CABG	7 (14.0)	7 (8.5)		4 (7.3)	1 (6.3)	-	-	0.69
AVR	0	0		1 (1.8)	0	-	-	0.60
Peripheral bypass	0	4 (4.9)		1 (1.8)	1 (6.3)	-	-	0.29
Operative time (min)	447.9 ± 167.6	452.3 ± 152.5		378.6 ± 97.9	379.2 ± 84.4	-	4.08	<0.01
CPB time	209.9 ± 77.7	221.2 ± 69.0		190.3 ± 47.5	196.2 ± 36.5	-	2.73	0.05
CA time	39.6 ± 19.4	41.2 ± 9.1		40.9 ± 9.9	40.6 ± 8.8	-	0.18	0.91
ACP (n, %)	24 (48.0)	56 (68.3)		43 (78.2)	11 (68.8)	11.10	-	0.01
ACP time	34.2 ± 47.8	52.8 ± 46.1		39.2 ± 35.2	36.0 ± 38.7	-	2.37	0.07
Rectal minimum temperature	21.7 ± 2.7	22.6 ± 3.0		23.8 ± 2.3	23.5 ± 2.4	-	5.61	<0.01
Intraoperative RBC units	12.4 ± 12.8 (n = 48)	11.3 ± 9.3 (n = 80)		6.2 ± 5.2 (n = 54)	3.5 ± 3.2 (n = 15)	-	7.61	<0.01
Intraoperative FFP units	18.1 ± 13.4 (n = 48)	18.5 ± 14.7 (n = 80)		16.7 ± 11.1 (n = 54)	18.8 ± 11.3 (n = 15)	-	0.28	0.88
Intraoperative platelets units	17.8 ± 10.3 (n = 48)	18.0 ± 10.8 (n = 80)		13.2 ± 7.3 (n = 54)	11.3 ± 2.7 (n = 15)	-	4.05	<0.01

Postoperative outcomes

The postoperative outcomes are summarized in Table 4.

**Table 4:** Early outcomes All values are expressed as n (%) and were compared using the Fisher’s exact test. A p-value < 0.05 was considered statistically significant. LCS-LCS: late-career surgeons as the primary surgeon and the first assistant; LCS-ECS: a late-career surgeon as the primary surgeon and an early surgeon as the first assistant; ECS-LCS: an early-career surgeon as the primary surgeon and a late-career surgeon as the first assistant; ECS-ECS: early-career surgeons as both primary surgeon and first assistant; CVD: cerebrovascular disease

Admission variables	LCS-LCS (n = 50)	LCS-ECS (n = 82)	ECS-LCS (n = 55)	ECS-ECS (n = 16)	p-value
Reoperation for bleeding (n, %)	2 (4.0)	5 (6.1)	4 (7.3)	0	0.83
New-onset CVD	6 (12.0)	11 (13.4)	5 (9.1)	2 (12.5)	0.89
Tracheostomy	7 (14.0)	9 (11.0)	7 (12.7)	1 (6.3)	0.90
Requiring transient dialysis	5 (10.2)	10 (12.5)	3 (5.6)	1 (6.7)	0.58
Requiring permanent dialysis	0	2 (2.5)	0	0	0.40
30-day mortality	3 (6.0)	6 (7.3)	2 (3.6)	1 (6.3)	0.79
In-hospital mortality	5 (10.0)	10 (12.2)	3 (5.5)	1 (6.3)	0.63

The unadjusted in-hospital mortality rates were 10.0%, 12.2%, 5.5%, and 6.3% for the LCS-LCS, LCS-ECS, ECS-LCS, and ECS-ECS groups, respectively (p = 0.63) (Table 4). In multivariate analysis, after correction for pre- and intraoperative factors, the primary surgeon’s years in practice were not a risk factor for in-hospital mortality. The significant risk factors for mortality were preoperative acute neurological complications (odds ratio (OR), 7.12; p = 0.01), operative time (OR, 1.01; p < 0.01), and concomitant procedures (OR, 4.84; p = 0.03). ATAAD repair cases per year performed by the first assistant was a protective factor (OR, 0.54; p = 0.02) (Table 5).

**Table 5:** Logistic regression showing predictors of in-hospital mortality Binomial logistic regression analysis for in-hospital mortality was performed on all pre- and intraoperative variables using a forward-backward stepwise approach based on a p-value of <0.1. The p-values were considered statistically significant at 0.05. ATAAD: acute type A aortic dissection

	Multivariable analysis		
Admission variables	Odds ratio	95% confidence interval	p-value
Age	1.06	0.99-1.12	0.06
Preoperative acute neurological complication	7.12	1.55-32.75	0.01
Preoperative malperfusion	3.96	0.76-20.74	0.10
Operative time	1.01	1.00-1.01	<0.01
Concomitant procedures	4.84	1.16-20.13	0.03
ATAAD repair cases/year of first assistant	0.54	0.33-0.90	0.02

**Figure 1:**
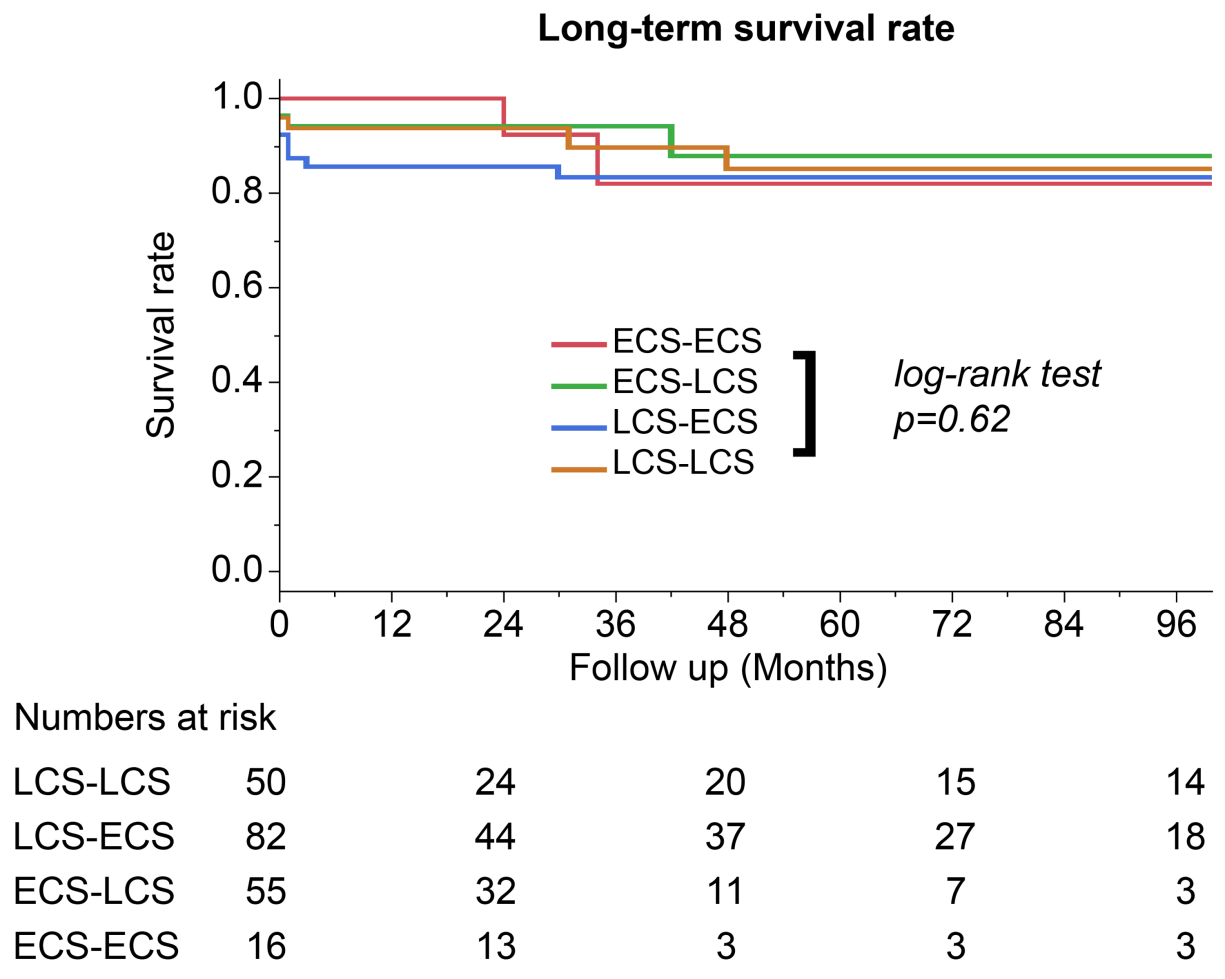
Kaplan-Meier eight-year survival curve with log-rank test comparing surgical teams LCS-LCS: late-career surgeons as the primary operator and the first assistant; LCS-ECS: a late-career surgeon as the primary surgeon and an early-career surgeon as the first assistant; ECS-LCS: an early-career surgeon as the primary surgeon and a late-career surgeon as the first assistant; ECS-ECS: early-career surgeons as both the primary surgeon and first assistant

**Figure 2:**
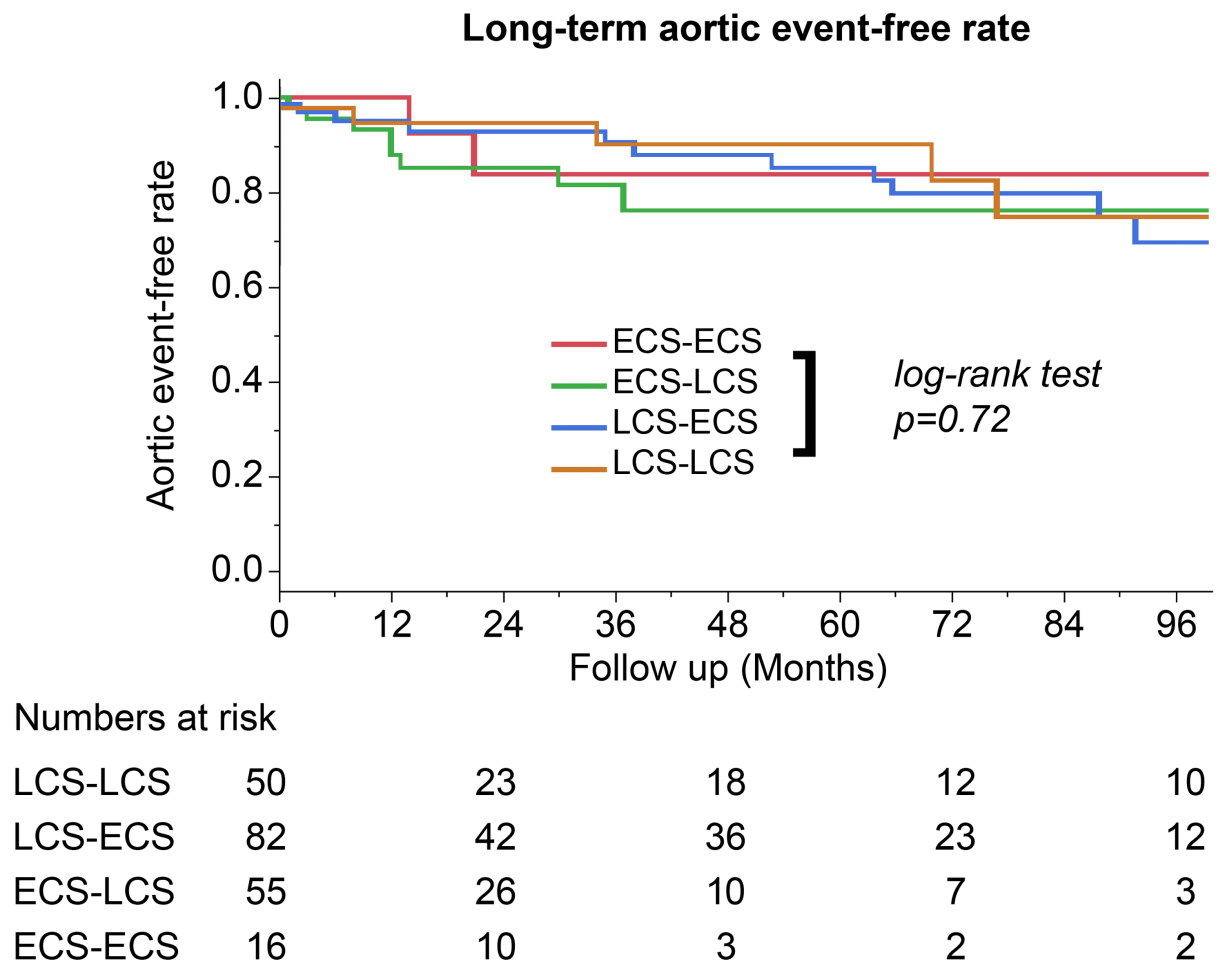
Kaplan-Meier eight-year aortic event-free curve with log-rank test comparing surgical teams LCS-LCS: late-career surgeons as the primary surgeon and first assistant; LCS-ECS: a late-career surgeon as the primary surgeon and an early surgeon as the first assistant; ECS-LCS: an early-career surgeon as the primary surgeon and a late-career surgeon as the first assistant; ECS-ECS: early-career surgeons as both the primary surgeon and first assistant

There were no significant differences in the rates of major complications, including reoperation, tracheostomy, and new onset cerebrovascular disease, between the groups. Furthermore, there was no significant difference in the long-term survival rates (p = 0.62) (Figure 1) and aortic event-free rates between the groups (p = 0.72) (Figure 2).

## Discussion

To the best of our knowledge, this is the first study to evaluate the effect of the surgeons’ years of practice as primary surgeons and as first assistants on outcomes of ATAAD repair. Although at least one LCS-included team may have been assigned to patients with a more severe status, the surgeons’ years of experience did not affect short- and long-term outcomes, nor was it a risk factor for in-hospital mortality in the multivariate analysis.

There have been many discussions on the relationship between surgeon experience and surgical results. However, which related parameter, such as surgeon's age, surgical volume, and years of clinical practice, is the best indicator of surgeon experience, remains undetermined. Previous studies have reported that surgeries performed by older surgeons have better surgical outcomes [3,5], whereas others have suggested the opposite [1,2]. However, it is important to note that a surgeon’s age may be biased due to various factors, such as training pathway and age at medical school admission [5,6,10], although Japanese surgeons’ age generally corresponds with their years in practice, as shown in our study (Table 1). Some studies have claimed that high-volume surgeons achieve better surgical results [7-9]. However, the method for determining the number of cases that would define a surgeon as “experienced” and which type of volume - number of recent operations, past operations, or total operations - is the most important factor remains unclear [7]. Recently, surgeons’ years in practice have gained popularity as a measure of their experience because it could assess their clinical experience more precisely [2,4,10]. While there are a few studies in the field of general surgery, this topic remains fully unexplored in the field of cardiac surgery, which involves high-risk surgeries that may warrant examination [10].

In cardiac surgery, one previous study evaluating the relationship between the surgeons’ years in practice and the outcomes of different cardiac surgeries performed by them reported that when compared with surgeons of longer years in practice, surgeons with fewer years in practice demonstrated worse outcomes in valve surgery; however, no difference was observed in coronary artery bypass surgery, even after adjusting for case volume [10]. This suggests that a surgeon's experience could affect the surgical outcomes of complex surgeries. ATAAD repair is a cardiac surgery that has the highest mortality rate [8,14]; therefore, we hypothesized that surgeons with more experience would have better surgical outcomes for ATAADs. However, our study demonstrated that the surgical results did not significantly differ between the groups. One plausible explanation for this is that the surgeons in this study may have acquired a minimum level of experience and skill in cardiac surgery. It is important to note that the ECSs in our study had <16 years of clinical experience (range, 9-15 years), which is equivalent to that of attending cardiac surgeons or cardiac surgery fellows. In the earlier report, ECSs were defined as those with <10 years of surgical experience, including significantly less experienced surgeons, with four to eight years of experience [10]. These criteria differ significantly from those of our study. Another difference is the number of cases experienced by a surgeon. Previous studies reported that surgeons with higher volumes have better surgical results, even with ATAAD repair [7,8]. Chikwe et al. [16] noted that surgeons with roughly two cases per year could experience lower mortality with ATAAD. In the present study, the mean volumes of ATAAD repairs performed by LCSs and ECSs as primary surgeons were over four ATAAD repairs per year and were not significantly different (Table 1). This may be the reason why no significant outcome differences were observed between the LCS and ECS operators. Furthermore, the highest mortality rate among the groups was 12.2% (from LCS-ECS) in our study. Considering that the reported mortality rate for ATAAD is 11.8%-25.1% [17-19], the outcomes for all groups observed in our study can be considered to be within the acceptable range.

We conducted an analysis that considered not only the experience of the primary surgeons but also that of the assistants in ATAAD repair. Since surgery is a team effort, many surgeons are aware that experienced assistants are necessary for complex and technical surgeries. However, there are few reports on the relationship between assistant’s experience and surgical outcomes. In a study that classified experienced (high-volume) and inexperienced (low-volume) surgeons by volume in ATAAD repair and compared surgical outcomes, the worst surgical outcomes were observed in the low-low group - inexperienced primary surgeon and assistant - followed by the combination of low-high, high-high, and high-low groups [7]. This may imply that the experience of not only the primary surgeon, but also that of the assistant affects surgical outcomes. In pediatric cardiac surgery, the presence of an experienced assistant can mitigate the shortcomings of inexperienced primary surgeons [11]. In our study, the multivariate analysis of this study showed that the assistant's ATAAD volume was a protective factor for mortality. This indicates that having a surgeon who has performed more ATAAD surgeries as the first assistant may lead to improved outcomes.

These findings may be useful for on-call coverage (and how surgeons should accumulate experience) in medium-sized institutions. Previous reports have shown that surgical outcomes are better in large centers [15,16,20]. This seems to be attributable to the fact that all surgeons and staff can accumulate cases. In our hospital, there were 12.7 ± 5.4 ATAAD repair cases per year; this number is not comparable with that in a large center. Our study demonstrated that there was no difference in ATAAD outcomes based on surgeons’ years in practice, indicating that a surgeon with a certain level of training can obtain the same acceptable outcomes, regardless of the number of years in practice and the combination of the primary surgeon and first assistant. Furthermore, the assistant's surgical volume was important for the outcome. Therefore, it may be possible to further improve outcomes by having experienced surgeons supporting the primary surgeons as the first assistant rather than only a specific surgeon performing surgery, and accumulating the roles of both the operator and assistant, regardless of a surgeon's years of experience in practice.

Limitations

The present study has several limitations. This study was conducted at a single facility in Japan; therefore, it is possible that the surgeons’ and facilities’ competencies do not fully reflect those of other surgeons and facilities. Furthermore, because of the relatively small sample size, the analysis may not have been powerful enough to detect more subtle differences in the results. Finally, our data included only patients who underwent ATAAD repair and excluded patients who died before surgery.

## Conclusions

The present study demonstrated that the surgeons’ years in practice did not affect the outcomes of ATAAD repair, regardless of the designation being the primary surgeon or the first assistant. A surgeon with a certain level of training can obtain the same acceptable outcomes, regardless of the number of years in practice and the combination of the experiences of the primary surgeon and first assistant. Furthermore, the assistant’s surgical volume was an important factor in the surgery outcome. These results may be useful for on-call coverage in medium-sized facilities. However, further studies evaluating larger data from multiple facilities throughout Japan are necessary for generalization.
